# Effects of an Exercise Programme on Functional Capacity, Body Composition and Risk of Falls in Patients with Cirrhosis: A Randomized Clinical Trial

**DOI:** 10.1371/journal.pone.0151652

**Published:** 2016-03-24

**Authors:** Eva Román, Cristina García-Galcerán, Teresa Torrades, Silvia Herrera, Ana Marín, Maite Doñate, Edilmar Alvarado-Tapias, Jorge Malouf, Laura Nácher, Ricard Serra-Grima, Carlos Guarner, Juan Cordoba, German Soriano

**Affiliations:** 1 Gastroenterology Department, Institut de Recerca-IIB Sant Pau, Hospital de la Santa Creu i Sant Pau, Barcelona, Spain; 2 Escola Universitària d’Infermeria EUI Sant Pau, Barcelona, Spain; 3 Physical Medicine and Rehabilitation Department, Hospital de la Santa Creu i Sant Pau, Barcelona, Spain; 4 Internal Medicine Department, Hospital de la Santa Creu i Sant Pau, Barcelona, Spain; 5 Cardiology Department, Hospital de la Santa Creu i Sant Pau, Barcelona, Spain; 6 Universitat Autònoma de Barcelona, Bellaterra, Cerdanyola del Vallès, Spain; 7 CIBERehd, Instituto de Salud Carlos III, Madrid, Spain; 8 Liver Unit, Internal Medicine Department, Hospital Vall d’Hebron, Barcelona, Spain; The Chinese University of Hong Kong, HONG KONG

## Abstract

Patients with cirrhosis often have functional limitations, decreased muscle mass, and a high risk of falls. These variables could improve with exercise. The aim was to study the effects of moderate exercise on functional capacity, body composition and risk of falls in patients with cirrhosis. Twenty-three cirrhotic patients were randomized to an exercise programme (n = 14) or to a relaxation programme (n = 9). Both programmes consisted of a one-hour session 3 days a week for 12 weeks. At the beginning and end of the study, we measured functional capacity using the cardiopulmonary exercise test, evaluated body composition using anthropometry and dual energy X-ray absorptiometry, and estimated risk of falls using the Timed Up&Go test. In the exercise group, cardiopulmonary exercise test showed an increase in total effort time (p<0.001) and ventilatory anaerobic threshold time (p = 0.009). Upper thigh circumference increased and mid-arm and mid-thigh skinfold thickness decreased. Dual energy X-ray absorptiometry showed a decrease in fat body mass (-0.94 kg, 95%CI -0.48 to -1.41, p = 0.003) and an increase in lean body mass (1.05 kg, 95%CI 0.27 to 1.82, p = 0.01), lean appendicular mass (0.38 kg, 95%CI 0.06 to 0.69, p = 0.03) and lean leg mass (0.34 kg, 95%CI 0.10 to 0.57, p = 0.02). The Timed Up&Go test decreased at the end of the study compared to baseline (p = 0.02). No changes were observed in the relaxation group. We conclude that a moderate exercise programme in patients with cirrhosis improves functional capacity, increases muscle mass, and decreases body fat and the Timed Up&Go time.

***Trial Registration*:** ClinicalTrials.gov NCT01447537

## Introduction

Patients with cirrhosis frequently present sarcopenia, a condition defined as a decrease in muscle mass and strength [1–4]. Sarcopenia impairs effort tolerance and health-related quality of life [5–8], may favor the development of hepatic encephalopathy [4], and determines poor prognosis [2,9]. Furthermore, impaired muscle strength and functional limitations may contribute to the predisposition to falls observed in cirrhotic patients, particularly in those with cognitive dysfunction [10,11].

Several studies have shown that physical exercise improves muscle mass and strength, functional impairment and health-related quality of life in various populations, such as older adults [12] and patients with chronic diseases, such as chronic heart failure [13], end-stage renal failure [14], and Parkinson’s disease [15]. Few studies, however, have assessed physical exercise in patients with cirrhosis, probably due to safety concerns [16–18].

In a previous randomized study in individuals with cirrhosis, we observed that exercise together with branched chain amino acids supplementation was not only safe but also improved functional capacity, muscle mass and health-related quality of life [19]. In this previous study, we assessed functional capacity and muscle mass using the 6-minute walk test and anthropometry, respectively. However, the effects of exercise can be evaluated using more precise techniques, such as the cardiopulmonary exercise test (CPET) to assess functional capacity [20,21] and dual energy X-ray absorptiometry (DXA) to evaluate body composition [1]. Nevertheless, few studies to date have analyzed the effects of exercise on CPET results in patients with cirrhosis [22], and to our knowledge, the changes in body composition after an exercise programme have not been studied by DXA in these patients.

Exercise has proven useful in preventing falls in the elderly [23] but it has not yet been assessed whether it can decrease the risk of falls in patients with cirrhosis.

The aim of the present study was to determine the effects of moderate physical exercise on functional capacity, body composition and risk of falls using CPET, DXA and the Timed Up&Go (TUG) test, respectively, in patients with cirrhosis.

### Patients and Methods

Between September 2011 and June 2012, we conducted a prospective, randomized study at Hospital de la Santa Creu i Sant Pau, a tertiary care hospital in Barcelona, Spain. The protocol conformed to the 1975 Declaration of Helsinki and Guidelines for Good Clinical Practice in Clinical Trials and was approved by the institution’s clinical research ethics committee (Comité Ético de Investigación Clínica [CEIC] at Hospital de la Santa Creu i Sant Pau). All patients received information concerning their participation in the study and signed an informed consent form. The protocol was registered at ClinicalTrials.gov (NCT01447537).

### Patients

We included 25 consecutive outpatients treated for cirrhosis. Cirrhosis was diagnosed by clinical, analytical and ultrasonographic findings or by liver biopsy. All patients had previous decompensations. Exclusion criteria were hospitalization for decompensation of cirrhosis in the previous month, variceal bleeding in the previous three months, previous variceal bleeding at any time without secondary prophylaxis of hemorrhage, large esophageal varices without primary prophylaxis, end-stage cirrhosis as shown by the Model for End-stage Liver Disease (MELD) >25, hepatocellular carcinoma or other neoplasia, active alcohol intake (in the previous 12 months), current overt hepatic encephalopathy, presence of ascites or edema, marked symptomatic comorbidities, contraindication for exercise, life expectancy of less than 6 months, and refusal to participate.

### Study design

The study was carried out by a multidisciplinary team consisting of two physiotherapists, three nurses, four hepatologists, one cardiologist, one sports medicine physician, one internal medicine specialist, and one medical student. Patients were randomized to either an exercise programme (n = 15) or to a relaxation programme (n = 10). Both programmes consisted of one-hour sessions, 3 days a week for 12 weeks under the supervision of physiotherapists. The relaxation programme was a sham intervention to control for a possible effect of attention and contact time with healthcare professionals [24]. All patients in both groups were evaluated at three visits: at baseline, at 12 weeks (end of study), and at 24 weeks (follow up). Patients were enrolled and randomized by the hepatologists (CG and GS). Randomization was performed using consecutively numbered opaque sealed envelopes. The study was designed with a different number of patients in each group because of limited availability of space and physiotherapists. The 15 patients in the exercise group were consecutively allocated into two subgroups: seven patients performed the exercise programme from 3 pm to 4 pm and 8 patients did so from 4 pm to 5 pm. Exercise was performed in the same room for both groups and was supervised by the same physiotherapist (CGG). The 10 patients in the relaxation group were supervised from 3 pm to 4 pm in a different room by another physiotherapist (TT).

In contrast with our previous study in which patients received branched chain amino acids in addition to the exercise programme [19], in the present trial we did not prescribe any specific nutritional intervention because our purpose was to determine the specific effects of the exercise programme. Patients from both groups were instructed to follow their usual diet throughout the study.

### Outcomes and end points

At baseline and at the end of the study, we evaluated functional capacity by cardiopulmonary exercise test (CPET), body composition by anthropometry and dual energy X-ray absorptiometry (DXA), and risk of falls by the Timed Up&Go (TUG) test. The main end points were changes in functional capacity and muscle mass, and the secondary end point was the risk of falls. We hypothesized that these parameters would improve as a result of the exercise programme [19,25].

### Clinical and analytical data

We recorded demographic and clinical parameters such as the etiology of cirrhosis, previous decompensations, Child-Pugh class, the MELD score, and pharmacological treatment. Physical examination and blood analysis were performed at baseline and at the end of the study. Abdominal ultrasound was also performed at baseline and at the end of the study to rule out hepatocellular carcinoma and the presence of ascites.

Patients were instructed to inform a team member of any potential adverse effect occurring during the 12 weeks of the programme or during the 12 weeks after the study ended. The incidence of side effects, complications of cirrhosis and hospitalizations during these periods were recorded.

### Anthropometric assessments

At baseline and at the end of the study, the same investigators (CGP in the exercise group and TT in the relaxation group) determined weight, height, body mass index, and circumference measurements in the right arm and leg to estimate muscle mass and fat mass. Leg measurements consisted of upper and lower thigh circumferences, measured at one third and at two thirds, respectively, of the line between the trochanter and the upper edge of the kneecap; and mid-thigh skinfold thickness. Arm measurements consisted of mid-arm circumference and mid-arm skinfold thickness. Mid-arm muscle circumference was calculated from these two measurements [26,27].

### Body composition by dual energy X-ray absorptiometry

Tissue composition analysis was performed by dual energy X-ray absorptiometry (DXA) using the Hologic Discovery DXA system® (HOLOGIC, Bedford, MA, USA). The coefficient of variation was 1%. Scan acquisition and analysis were performed blindly by certified and experienced technicians (SH and AM) in accordance with ISCD standards (http://www.iscd.org/documents/2015/06/2015-iscd-adult-official-positions.pdf).

We specifically determined fat body mass, lean body mass, lean appendicular mass and lean leg mass. As no patient presented ascites or edema, lean mass was considered an index of muscle mass.

### Functional capacity by cardiopulmonary exercise test

At the beginning and end of the study, participants performed a stress test on a Schiller treadmill STM–55/65 model using a ramp protocol [28,29]. The supervisors of cardiopulmonary exercise test (CPET) (MTD and RSG) were blinded to the group the patients had been assigned to. The initial treadmill speed was 3 km/h for the first 2 minutes, increasing 0.3 km/h for each subsequent minute. The initial slope was zero for the first 2 minutes, increasing 1.4% for each subsequent minute until it reached a maximum of 12%. Twelve-lead ECG (CS-200) recordings were made and blood pressure was taken using a Riester sphygmomanometer. A mask was used to collect exhaled gases. Using a Ganshorn Power-Cube gas analyser, we determined the following parameters: oxygen uptake (VO_2_) in ml·kg·min, oxygen pulse (PO_2_) in ml/beats, carbon dioxide production (VCO_2_) in l/min, respiratory quotient (RQ), baseline heart rate (HR) and maximum HR under stress (stress HR), ventilatory anaerobic threshold (VAT) expressed in relation to HR, percentage of VO_2_ achieved in the VAT (%VO_2_), and exhaled volume in l/min. Baseline and maximum blood pressure were determined at each stage, and test duration was noted in minutes. The criteria for ending the test were a lack of increase in VO_2_, physical exhaustion, or the patient’s request to stop the test. Participants were considered to have reached the maximum when a VO_2_ plateau appeared, or when the VAT appeared if the former was lacking.

### Timed Up&Go test

The Timed Up&Go (TUG) test evaluates physical performance [1] and has been used in previous studies to estimate the risk of falls [11,30]. This test measures the time that the patient needs to stand up from a chair, walk three meters, come back, and sit down again without help [30].

### Physical exercise programme

Patients in the exercise group undertook a 12-week exercise programme, similar to programmes used in other populations, such as patients with chronic heart disease or respiratory disease [31,32], and to that used in patients with cirrhosis in our previous study [19]. The programme was conducted at the hospital and consisted of 36 one-hour sessions, held on Mondays, Wednesdays and Fridays. Patients were divided into 2 subgroups and were always led by a physiotherapist (CGG). We measured oxygen saturation, heart rate and blood pressure before, during and at the end of each exercise session. The patient’s attendance at each session was recorded.

Exercise consisted mainly of cycle ergometry and treadmill walking. After a 10-minute warm up, cycle ergometry combined with treadmill walking was performed for 10–15 minutes per session at the beginning of the programme and steadily increased to 25–30 minutes at the end of the programme. Initial treadmill velocity was calculated and later increased according to patient tolerance. Patients also performed resistance exercise for arms using free weights and elastic bands for 5–10 minutes. At the end of each session, patients performed balance, coordination, stretching and relaxation exercises for 10–15 minutes. The intensity of exercise was increased on the basis of patient tolerance. To prevent variceal bleeding, the programme did not include exercises involving the abdominal muscles or exercises that could increase intra-abdominal pressure. The intensity of the programme was considered moderate, as it was planned that patients worked at 60–70% of the maximum heart rate at baseline CPET [33].

### Relaxation programme

The relaxation programme consisted of 36 one-hour sessions held on Mondays, Wednesdays and Fridays over 12 weeks. Patients in this programme were included in a single group of 10 patients. Sessions were led by a physiotherapist trained in relaxation techniques based on sophrology (TT); they included cephalocaudal muscle relaxation, and breathing, visualization and concentration exercises [34].

### Statistical analysis

Baseline characteristics of patients in both groups were compared using Fisher’s exact test for categorical variables and Student’s t test or Mann-Whitney test for quantitative variables. We used the Student’s t test when data presented a normal distribution and the Mann-Whitney test when data did not present normal distribution. Normality was assessed by the Shapiro-Wilk test.

To evaluate variations at the end of the study with respect to baseline values in each group, we used the paired Student’s t test or the Wilcoxon test, depending on whether the data distribution was normal or non-normal, respectively. Correlations were assessed using Pearson’s test. Results are expressed as mean±standard error of the mean, % differences with 95% confidence intervals (CI), or frequencies and percentages. A two-sided p value of p<0.05 was considered statistically significant.

The sample size was calculated using previous data on changes in exercise capacity after exercise in patients with cirrhosis [19]. Considering a 30% increase in exercise capacity with an estimated standard deviation of 30%, an alpha error of 0.05, a power of 0.80 and 20% of lost patients, the minimal number of patients needed to detect a significant increase in exercise capacity after the exercise programme was 10.

Calculations were performed using the SPSS Statistical Package (version 21.0, 2012; SPSS Inc., Chicago, IL).

## Results

**Fig 1:**
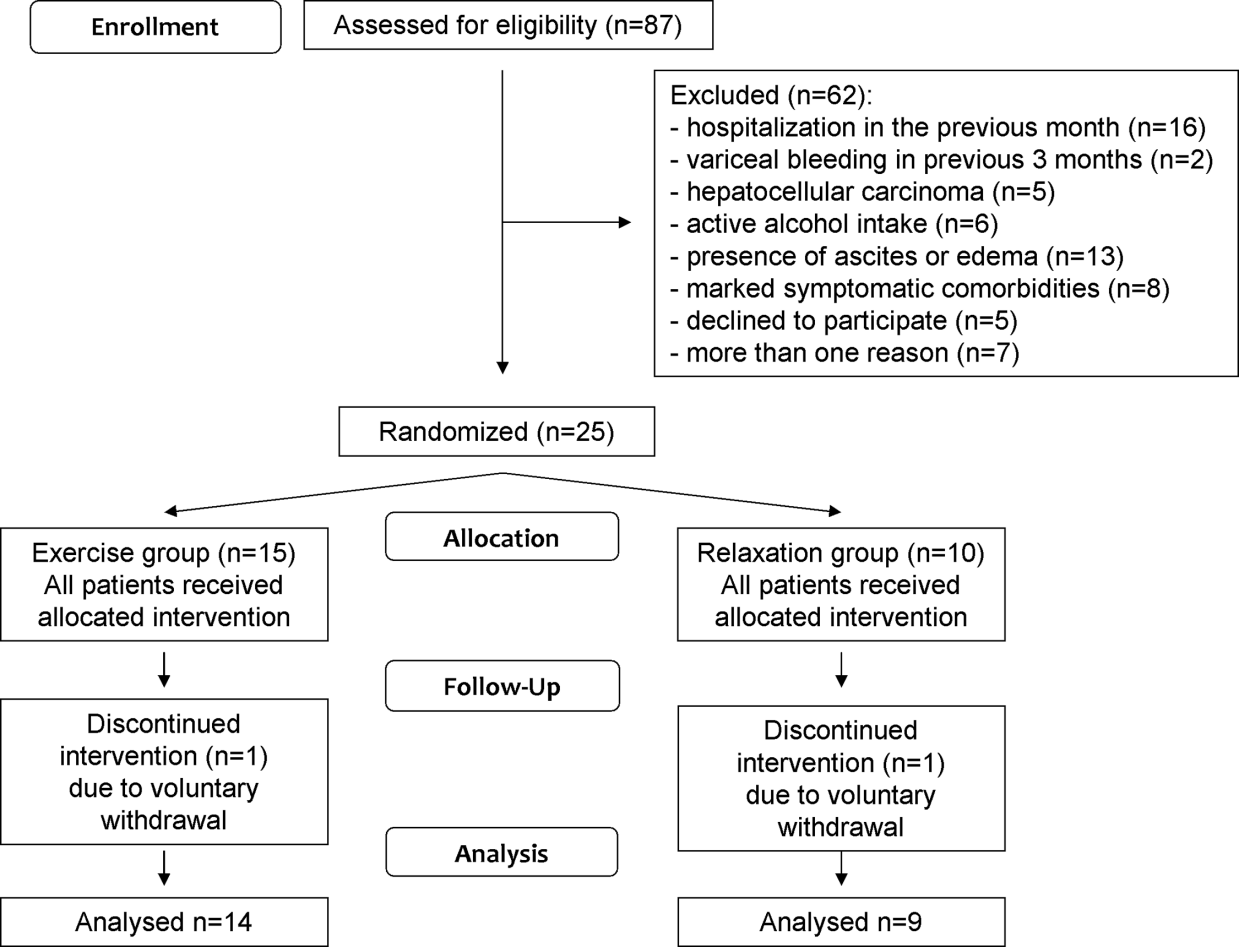
Flowchart of the study.

We evaluated 87 outpatients with cirrhosis and 62 were excluded for several reasons (see CONSORT flowchart in **Fig 1**). Of the 25 patients included in the study, one patient in the exercise group and one patient in the relaxation group did not complete the programme due to voluntary withdrawal. Final analysis therefore included 14 patients in the exercise group and 9 patients in the relaxation group. The mean number of sessions missed was 2.3±0.6 in the exercise group and 12.6±4.2 in the relaxation group (p = 0.004).

### Patients’ clinical and analytical characteristics

Table 1 shows that baseline characteristics were similar in the two groups. There were no differences in demographic data, etiology of cirrhosis, degree of liver insufficiency, previous complications, pharmacological treatment, renal function, or body mass index.

**Table 1:** Clinical and analytical characteristics of patients in each group. Results are expressed as mean±standard error of the mean or frequencies (%).

	Exercise group (n = 14)	Relaxation group (n = 9)
Age (years)	62.0±2.4	63.1±2.3
Gender (male/female)	10 (71%)/4 (29%)	7 (78%)/2 (22%)
Child-Pugh score	5.4±0.2	5.4±0.2
MELD^a^ score	8.2±0.4	9.1±0.4
Etiology (alcohol/virus/other)	12 (86%)/1 (7%)/1 (7%)	5 (56%)/2 (22%)/2 (22%)
Previous decompensation of cirrhosis	14 (100%)	9 (100%)
Previous ascites	12 (86%)	5 (56%)
Previous encephalopathy	1 (7%)	1 (11%)
Previous variceal bleeding	2 (14%)	3 (33%)
Beta-blockers	5 (36%)	6 (67%)
Bilirubin (μmol/L)	18.1±2.8	24.1±2.6
Albumin (g/L)	37.6±1.1	36.2±1.5
INR^b^	1.14±0.04	1.15±0.04
Creatinine (μmol/L)	77.2±5.5	72.2±4.4
Body mass index (kg/m^2^)	31.5±1.6	30.3±1.4
Obesity (body mass index ≥ 30 kg/m^2^)	10 (66.7%)	5 (55.6%)

^a^MELD: Model for End-stage Liver Disease.

^b^INR: international normalized ratio.

There were no statistically significant differences between the two groups.

### Anthropometric measurements

Table 2 shows the changes in anthropometric parameters. Patients in the exercise group increased upper thigh circumference (51.1±2.0 cm at baseline to 55.3±2.3 at the end of the study, difference 4.25 cm, 95%CI 1.62 to 6.87, p = 0.009) and decreased mid-arm circumference (34.1±1.3 cm to 33.5±1.3, difference -0.60 cm, 95%CI -0.12 to -1.08, p = 0.02), mid-arm skinfold thickness (25.1±1.6 mm to 21.9±1.5, difference -3.14 mm, 95%CI -0.81 to– 5.47, p = 0.01) and mid-thigh skinfold thickness (30.8±1.6 mm to 27.0±1.6, difference -3.82 mm, 95%CI -1.10 to -6.53, p = 0.01). No statistically significant changes were observed in the remaining anthropometric parameters in the exercise group, including weight or mid-arm muscle circumference, or in any of these parameters in the relaxation group.

**Table 2:** Changes in the anthropometric measurements in patients in each group. Results are expressed as mean±standard error of the mean.

	Exercise group		Relaxation group^a^	
	Baseline (n = 14)	12 weeks (n = 14)	Baseline (n = 8)	12 weeks (n = 8)
Weight (kg)	83.8±5.1	83.7±5.4	80.7±4.1	80.4±3.7
Upper thigh circumference (cm)	51.1±2.0	55.3±2.3^b^	54.8±1.3	54.6±1.6
Lower thigh circumference (cm)	47.8±2.1	48.3±2.3	46.3±1.3	45.0±1.7
Mid-arm circumference (cm)	34.1±1.3	33.5±1.3^c^	29.4±2.9	29.0±2.6
Mid-arm skinfold thickness (mm)	25.1±1.6	21.9±1.5^d^	19.0±2.0^f^	19.1±2.0
Mid-arm muscle circumference (cm)	26.2±1.0	26.6±1.0	23.8±2.5	23.5±2.3
Mid-thigh skinfold thickness (mm)	30.8±1.6	27.0±1.6^e^	28.8±2.1	28.0±2.3

^a^Data not available for one patient in the relaxation group.

^b^p = 0.009

^c^p = 0.02

^d^p = 0.01 and

^e^p = 0.01 vs baseline in the exercise group.

^f^p = 0.03 with respect to baseline in the exercise group.

No other statistically significant differences were found between groups at baseline or at 12 weeks or between time points in each group.

### Body composition by dual energy X-ray absorptiometry

Body composition was evaluated by dual energy X-ray absorptiometry (DXA) at baseline in all 23 patients. Three patients (13%, 2/14 in the exercise group and 1/9 in the relaxation group) presented sarcopenia according to the skeletal muscle mass index (appendicular muscle mass/height^2^) reference values [1].

**Fig 2:**
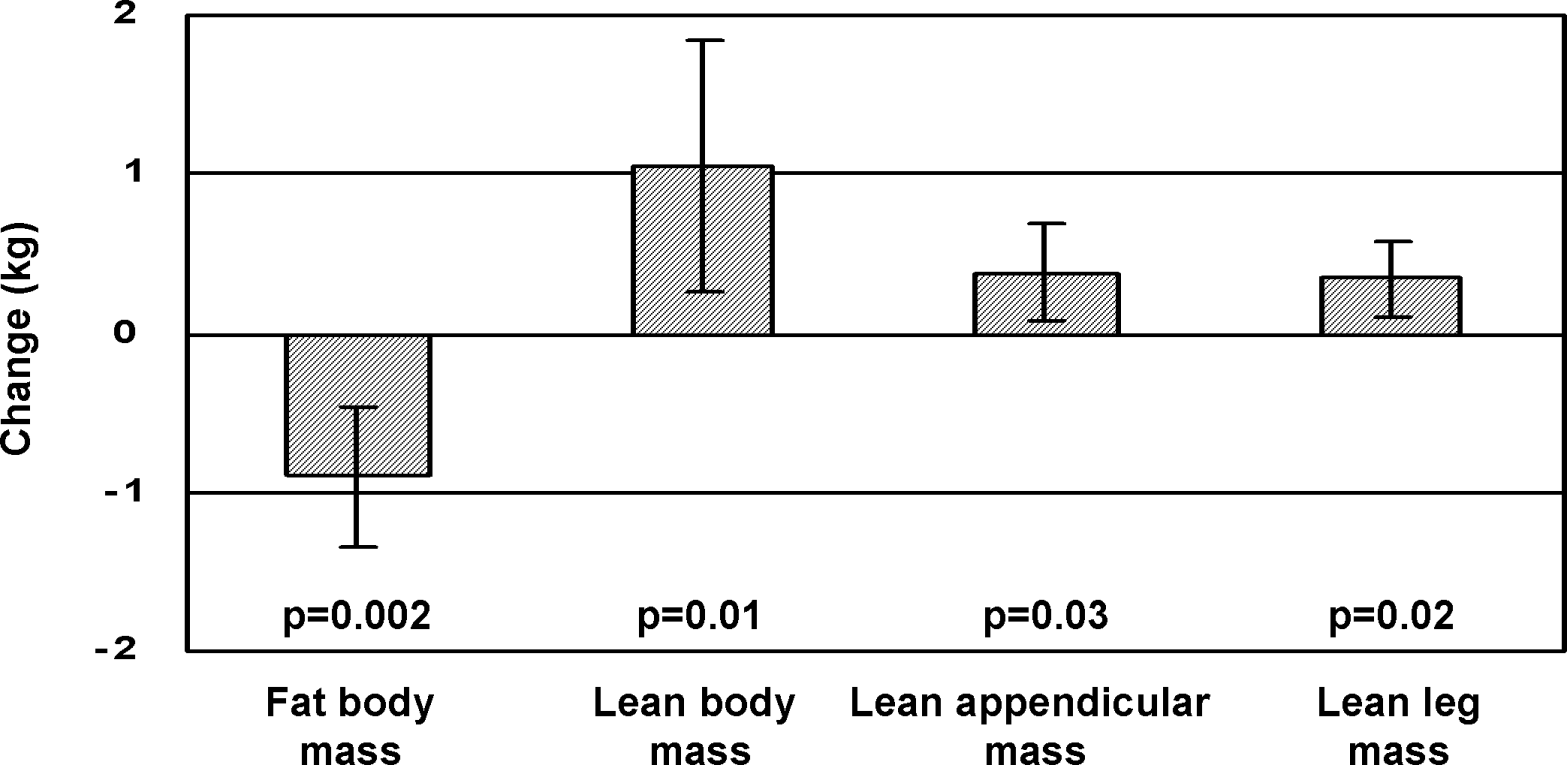
Changes in body composition by dual energy X-ray absorptiometry (DXA) between baseline and end of the study in patients from the exercise group. Results are expressed as difference and 95%CI.

DXA was performed at the end of the study in 22 patients (13 in the exercise group and 9 in the relaxation group). We did not find statistically significant differences between the two groups in body composition by DXA at baseline or at the end of the study. **Fig 2**shows the changes in body composition by DXA in the exercise group. We observed a decrease in fat body mass (29.18±2.64 kg at baseline to 28.58±2.88 kg at the end of the study, difference -0.94 kg, 95%CI -0.48 to -1.41, p = 0.003) and an increase in lean body mass (48.19±3.06 to 49.25±3.19 kg, difference 1.05 kg, 95%CI 0.27 to 1.82, p = 0.01), lean appendicular mass (21.36±1.64 to 21.74±1.63 kg, difference 0.38 kg, 95%CI 0.06 to 0.69, p = 0.03) and lean leg mass (16.72±1.48 to 17.06±1.49 kg, difference 0.34 kg, 95%CI 0.10 to 0.57, p = 0.02). No statistically significant changes were observed between baseline and the end of the study in the relaxation group: fat body mass 0.21 kg, 95%CI -1.05 to 1.47 (p = 0.67), lean body mass -0.05 kg, 95%CI -1.25 to 1.15 (p = 0.76), lean appendicular mass -0.17 kg, 95%CI -0.76 to 0.41 (p = 0.51), and lean leg mass -0.14 kg, 95%CI -0.59 to 0.30 (p = 0.67).

**Fig 3:**
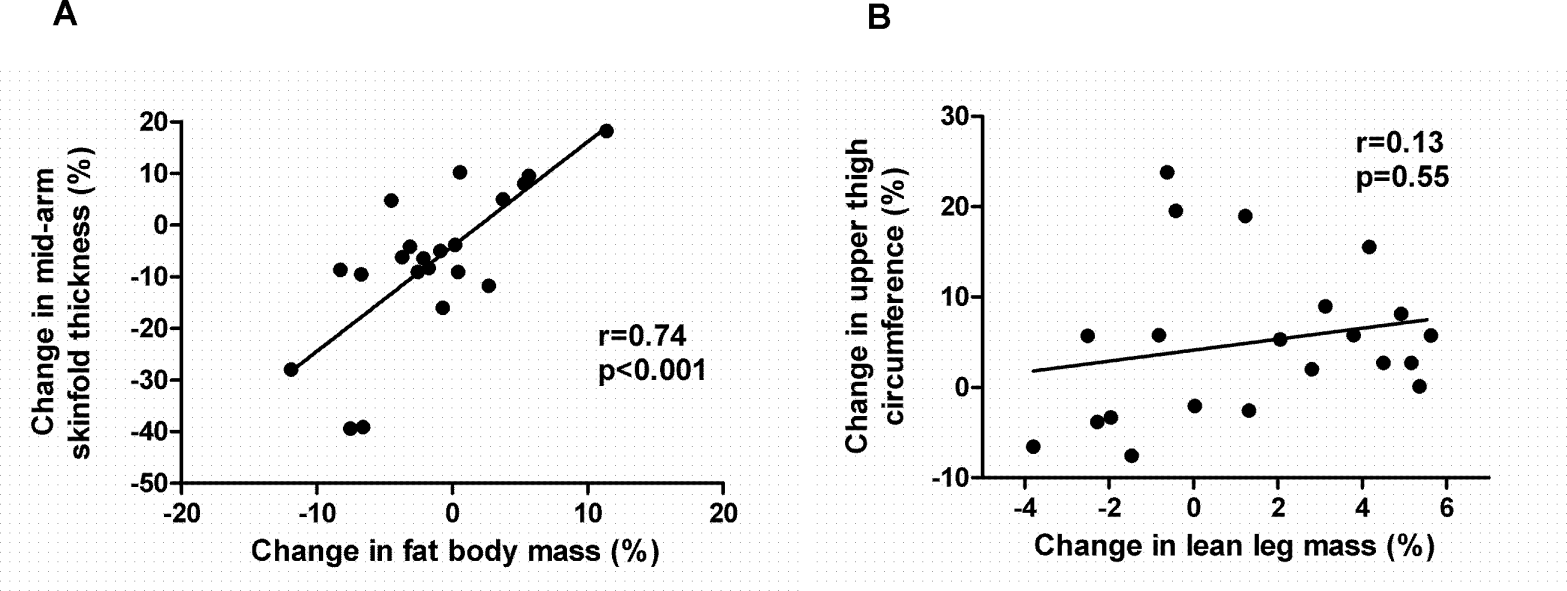
Correlations between changes in anthropometric measurements and changes in body composition assessed by dual energy x-ray absorptiometry (DXA): (A) mid-arm skinfold thickness and fat body mass, (B) upper thigh circumference and lean leg mass. Results are expressed as % change.

When comparing DXA with anthropometric measurements in all patients in the study, we observed a direct correlation between the change in fat body mass and the change in mid-arm skinfold thickness (r = 0.74, p<0.001) (**Fig 3A**). However, there was no correlation between the change in lean leg mass and the change in upper thigh circumference (r = 0.21, p = 0.36) (**Fig 3B**).

### Functional capacity by cardiopulmonary exercise test

Cardiopulmonary exercise test (CPET) was performed at baseline in 22 patients (14 in the exercise group and 8 in the relaxation group). Fifteen patients (68.1%, 10/14 in the exercise group and 5/8 in the relaxation group) presented functional limitations evaluated by VO_2peak_ according to reference values adjusted for age and gender.

Table 3 shows the changes in CPET. Patients in the exercise group increased total effort time (8.5±0.6 to 10.5±0.6 min, difference 2.00 min, 95%CI 1.16 to 2.83, p<0.001) and ventilatory anaerobic threshold (VAT) time (6.6±0.5 to 8.1±0.3 min, difference 1.50 min, 95%CI 0.57 to 2.42, p = 0.009). Peak oxygen uptake (VO_2peak_) increased but did not reach statistical significance. The number of patients with functional limitations evaluated by VO_2peak_ according to reference values changed from 10/14 (71.4%) at the beginning of the study to 6/14 (42.8%) at study end (p = 0.25). Four patients in the relaxation group did not perform CPET at the end of the study for different reasons: one had not performed the test at baseline, one was diagnosed of hepatocellular carcinoma, one was diagnosed of breast adenocarcinoma, and one missed many sessions and preferred not to perform the CPET. The remaining 5 patients in the relaxation group did not show any statistically significant changes in CPET parameters.

**Table 3:** Changes in functional capacity evaluated by cardiopulmonary exercise test (CPET) in patients in each group at baseline and at the end of the study (12 weeks). Results are expressed as mean±standard error of the mean or frequencies (%).

	Exercise group		Relaxation group	
	Baseline (n = 14)	12 weeks (n = 14)	Baseline (n = 8)	12 weeks^a^ (n = 5)
Total effort time (min)	8.5±0.6	10.5±0.6^d^	9.3±1.0	11.0±1.3
VAT^b^ time (min)	6.6±0.5	8.1±0.3^e^	5.1±1.2	7.5±1.7
Maximal heart rate (bpm)	129.1±5.0	137.9±3.5^f^	123.3±7.0	110.2±6.5
VO_2peak_^c^/kg weight (ml/kg/min)	21.4±0.8	23.0±1.3	23.5±2.9	25.0±3.2
Functional limitation evaluated by VO_2peak_	10/14 (71.4%)	6/14 (42.8%)	5/8 (62.5%)	3/5 (60%)

^a^Only 5 patients were evaluated by CPET at 12 weeks.

^b^VAT: ventilatory anaerobic threshold.

^c^VO_2peak_: peak oxygen uptake.

^d^p<0.001

^e^p = 0.009 vs baseline in the exercise group.

^f^p = 0.01 vs baseline in the exercise group, and p = 0.001 vs 12 weeks in the relaxation group.

No other statistically significant differences were observed between groups at baseline or at 12 weeks, or between time points in each group.

### Timed Up&Go test

**Fig 4:**
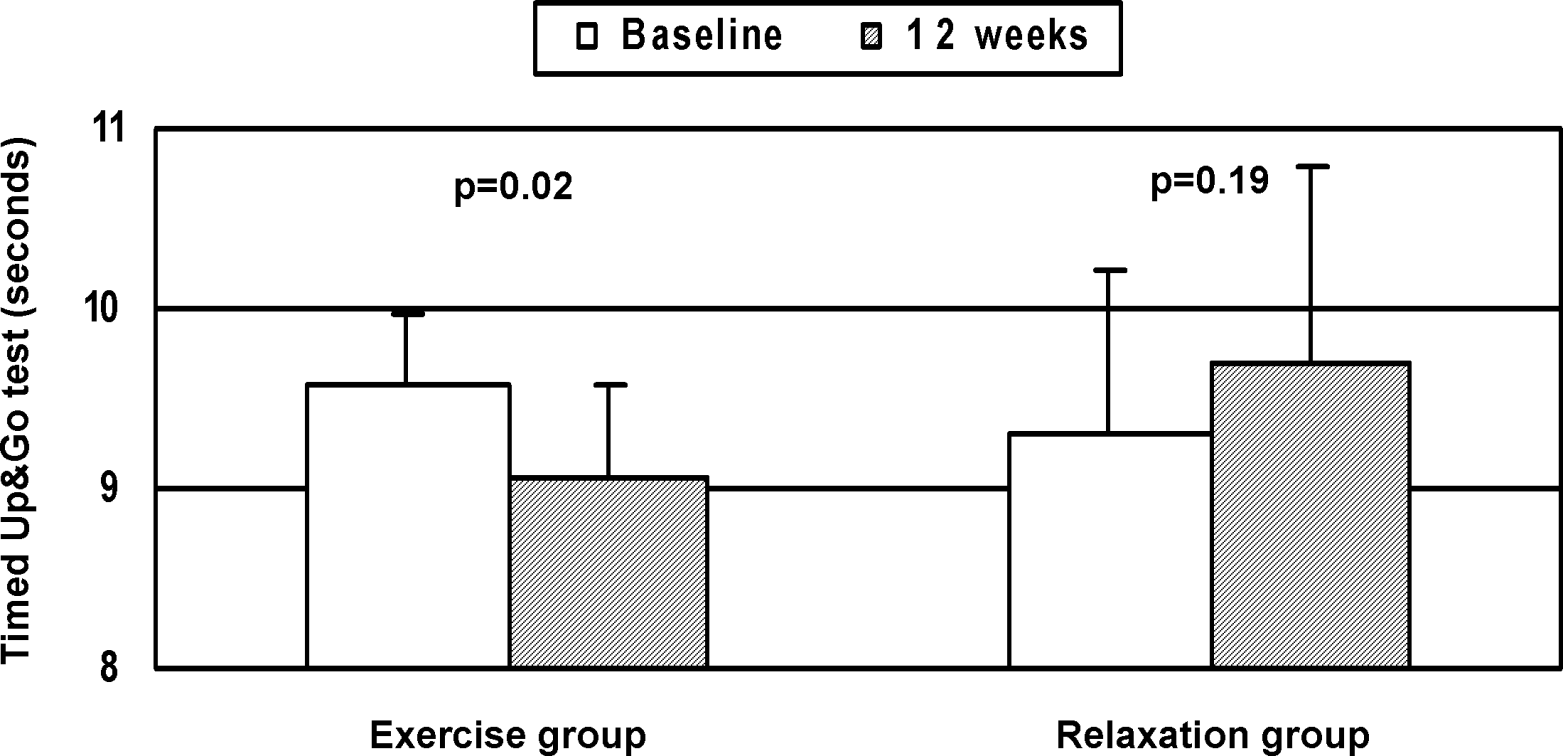
Risk of falls evaluated using the Timed Up&Go test at baseline and at the end of the study (12 weeks) in both groups. Results are expressed as mean±standard error of the mean.

**Fig 4**shows the results of the Timed Up&Go (TUG) test at baseline and at the end of the study in the 23 participants. In the exercise group, TUG decreased at the end of the study with respect to baseline (9.6±0.4 sec vs 9.1±0.4, difference -0.50 sec, 95%CI -0.12 to -0.87, p = 0.02). In the relaxation group, there were no statistically significant differences between baseline and 12-week measurements. There were no statistically significant differences between the two groups at baseline or at 12 weeks.

### Safety

One patient in the exercise group, previously diagnosed of asthma, presented a mild bronchospasm during the exercise programme. He did not require hospitalization but was unable to attend 9 of the sessions. In the relaxation group, one patient was diagnosed of hepatocellular carcinoma during the study and another patient was diagnosed of breast adenocarcinoma. No decompensations of cirrhosis were diagnosed during the study or during the follow-up period in either group.

## Discussion

In the present study we observed that a moderate exercise programme evaluated by CPET and DXA improved exercise capacity, increased muscle mass and decreased fat mass in patients with cirrhosis.

In a previous study, we found that a moderate exercise programme associated with branched chain amino acids supplementation increased effort tolerance evaluated by the 6-minute walk test, muscle mass estimated by anthropometric measurements, and health-related quality of life assessed using the SF-36 questionnaire in cirrhotic patients [19]. In the present study, we aimed to further investigate the effects of exercise in these patients. We evaluated an exercise programme alone and used more precise techniques than those used in our previous study.

The observed increase in total effort time and VAT time on CPET in the exercise group suggests an improvement in functional capacity. Although the improvement in VO_2peak_ did not reach statistical significance, the percentage of patients with a functional limitation considering reference VO_2peak_ values decreased from 71.4% at baseline to 42.8% at the end of the study. Zenith et al [20] and Debette-Gratien et al [21] recently described a statistically significant increase in VO_2peak_ values in cirrhotic patients after an 8-week and 12-week exercise programme, respectively. The lack of statistical significance in the increase in VO_2peak_ in the present study could be because our patients were older and had a higher body mass index, and also because the exercise programme differed from that in the two previous studies [20,21]. Differences in the type of exercises, in the duration of the sessions, in the number of patients per session (two patients in the study of Debette-Gratien et al [21] vs 7 patients in the present study), and especially in the intensity of the exercise (60–70% of the maximum heart rate in the present study vs higher intensities in the two previous studies [20,21]) could partly account for this discrepancy.

To our knowledge, this is the first study to analyze the effects of exercise on body composition in patients with cirrhosis using DXA. DXA results indicated an increase in muscle mass and a decrease in fat mass in patients in the exercise group. One limitation with the use of DXA to evaluate body composition in patients with cirrhosis is the potential interference of fluid retention when muscle mass is extrapolated from lean mass results [3]. However, our patients were in a stable condition and fluid retention was not detected in any patient by physical and ultrasound examination during the study. Our results are in line with those in the recently reported randomized controlled trial by Zenith et al [20]. These authors observed an increase in thigh muscle thickness measured by ultrasound in the 9 patients with stable cirrhosis after 8 weeks of exercise compared to the 10 patients in the control group.

The changes in anthropometric measures, an increase in upper thigh circumference and a decrease in skinfold thickness, also suggest an increase in muscle mass and a decrease in fat mass [26,27]. However, while there was a positive correlation between anthropometry and DXA in the evaluation of changes in fat body mass, we did not observe such a correlation in the evaluation of changes in muscle mass. This lack of correlation has been noted by other authors [1] and suggests that although anthropometric measures are more accessible and cost-effective in daily clinical practice than DXA, they are insensitive to subtle changes such as those observed in the present study. It is striking that in our study the circumference increased in the leg but decreased in the arm. The fact that patients performed most exercises with the legs and the loss of fat mass would explain this difference.

Sarcopenic obesity is described as a decrease in lean body mass with concomitant excess fat body mass, and it is increasingly recognized in older people and several chronic diseases, including cirrhosis [1,3]. In our study, more than half of the patients in both groups were obese. Those in the exercise group showed no changes in body weight despite gaining about one kilogram of muscle mass, probably due to the concomitant decrease in fat body mass of approximately one kilogram. Such a decrease is an interesting target considering that overweight and metabolic syndrome have been found to worsen prognosis in cirrhosis [35]. Berzigotti et al [36] recently reported that an exercise programme associated with a nutritional intervention in obese cirrhotic patients decreases body fat and improves portal hemodynamics.

Falls and fractures are frequent in patients with cirrhosis and a common cause of complications, hospitalizations and deterioration in health-related quality of life [10,11,37,38]. In the present study, the improvement in the TUG test at the end of the programme in the exercise group indicates not only an improvement in the functional capacity of patients [1], but also a decrease in the risk of falling [30]. This finding supports the hypothesis that exercise can help reduce the incidence of falls in patients with cirrhosis, as observed in other populations [23], but a prospective randomized clinical trial with a high number of patients is needed to confirm this hypothesis.

Regarding safety, our findings confirm the results from our previous study [19] as patients had no complications of cirrhosis during the development of the programme. These results are also in agreement with the studies by Zenith et al [20] and Debette-Gratien et al [21]. However, most patients included in these studies, even with previous decompensations, were in a stable situation at the beginning of the exercise programme. Caution should be taken into account here as exercise may worse portal hypertension in patients not taking beta-blockers [16,17] and renal function in patients with ascites [18]. Moreover, Berzigotti et al [22] recently underlined the need for nutritional assessment and supplementation when indicated before initiating an exercise programme in patients with decompensated cirrhosis to avoid further protein catabolism and loss of muscle mass.

In agreement with previous studies [19–21], the acceptability and compliance of the exercise programme was considered satisfactory. Patients in this group missed a mean of only 2.3 sessions. In contrast, in the relaxation group participants missed a higher number of sessions (12.6). We attribute this difference to a higher commitment and satisfaction of patients with the exercise than with the relaxation programme.

Our study has several limitations. The first of these is the small sample size. Nevertheless, such studies are difficult to conduct in a large number of patients due to limited availability of physiotherapists and shortage of space [19–21]. Moreover, most changes observed in the exercise group achieved statistical significance in spite of the small number of patients. Second, the low compliance with the programme and the study procedures, mainly CPET, among patients from the relaxation group limit the interpretation of the data. However, we did not observe significant changes in the parameters evaluated in an intention-to-treat analysis in this group, originally conceived as a control group. Finally, although more than two thirds of patients in our study presented at baseline functional limitations according to the results of the CPET, only 13% fulfilled the criteria for sarcopenia using the skeletal muscle mass index determined by DXA. This percentage is lower than the 40% previously reported incidence of sarcopenia in cirrhotic patients evaluated for liver transplantation using computed tomography scan at the third lumbar vertebrae [2]. Probably this difference can be due to the different methods used to evaluate sarcopenia but also to the fact that our study population included compensated outpatients with more preserved liver function. Studies with a larger number of patients with cirrhosis and sarcopenia are needed to assess the effects of exercise in this setting.

In conclusion, with the limitations of the small sample size, in this study using CPET and DXA we found that a moderate exercise programme increases effort tolerance and muscle mass, and decreases body fat and risk of falls estimated by the TUG test in patients with cirrhosis.

## Supporting Information

S1 Table The anonymized database with all the individual data of patients included in the study. (CSV)

S1 Text The explanation of the structure of the database. (DOC)

S2 Text The original protocol in English. (PDF)

S3 Text The original protocol in Spanish. (PDF)

S4 Text The approval of the original protocol by the Ethics Committee of our centre. (PDF)

S5 Text CONSORT checklist. (PDF)
