# Supplementary material for: Effects of an Exercise Programme on Functional Capacity, Body Composition and Risk of Falls in Patients with Cirrhosis: A Randomized Clinical Trial
Source: PLoS One. 2016 Mar 24;11(3):e0151652. doi: 10.1371/journal.pone.0151652 (PMC4807034; doi:10.1371/journal.pone.0151652)
Supplement: S1 Text — (DOC) [file pone.0151652.s002.doc]

**DATA STRUCTURE**

Most of the parameters are numeric values, except for sex (0=male, 1=female), etiology (1=alcohol, 2=virus, 3=other), and obesity, previous decompensation, previous encephalopathy, previous ascites, previous variceal bleeding, betablockers, functional limitation according to VO and sarcopenia according to DXA (for all these parameters 0=no, 1=yes).

Parameters that end with “2” (like “Timed_Up_and_Go_test2” or “Effort_time2”) refer to values at the end of the study (12 weeks) while parameters without “2” (like “Timed_Up_and_Go_test” or “Effort_time”) refer to baseline values.
