# Supplementary material for: Effects of an Exercise Programme on Functional Capacity, Body Composition and Risk of Falls in Patients with Cirrhosis: A Randomized Clinical Trial
Source: PLoS One. 2016 Mar 24;11(3):e0151652. doi: 10.1371/journal.pone.0151652 (PMC4807034; doi:10.1371/journal.pone.0151652)
Supplement: S2 Text — (PDF) [file pone.0151652.s003.pdf]

MECHANISMS INVOLVED IN THE BENEFITS OF AN EXERCISE PROGRAMME IN PATIENTS WITH CIRRHOSIS

CODE: 59/2010

Eva Román, Cristina García-Galcerán, Teresa Torrades, Silvia Herrera, Ana Marín, M. Teresa Doñate, Jorge Malouf, Laura Nácher, Ricard Serra-Grima, Carlos Guarner, Juan Cordoba and German Soriano

Department of Gastroenterology, Department of Physical Medicine and Rehabilitation, Department of Cardiology, Department of Immunology. Department of Internal Medicine. Hospital de la Santa Creu i Sant Pau. Barcelona. Spain.

Abstract

Background: The results of a previous pilot study including 17 patients with cirrhosis have shown that a moderate exercise programme for three months improves exercise tolerance and increases muscle mass without observed undesirable effects. In this previous study, however, we evaluated exercise tolerance and muscle mass by rough parameters, such as 6-minute walk test and anthropometry. We do not know the exact mechanisms involved in the beneficial effects of exercise in these patients.

Main aims:

1) To more accurately evaluate the effect of exercise on effort capacity, muscle mass, and inflammatory response in patients with cirrhosis.
2) Confirm the efficacy and safety of an exercise program in a larger series of patients with cirrhosis.

Subjects: Thirty patients with compensated liver cirrhosis.

Intervention: Patients will be randomized into two groups: One group will perform an exercise programme for 12 weeks, three days per week, and the other will be the control group that will perform a relaxation programme.

Evaluation: Clinical assessment and laboratory tests at the beginning and at 12 weeks (end of study). We will analyze changes in muscle mass using advanced techniques (densitometry, CT scan) in addition to anthropometry and in exercise tolerance by cardiopulmonary exercise test (CPET). We will also evaluate quality of life, glutamine synthetase activity, oxidative damage and inflammatory response. Complications of cirrhosis will be assessed during the study and follow-up.

Introduction

A high proportion of cirrhotic patients have decreased muscle mass, fatigue, poor exercise tolerance and reduced quality of life (1-5), impairing their normal activity. It has been suggested that these parameters may improve with moderate exercise (1), but some studies warned about the possible negative effects of acute exercise on renal function and portal pressure in patients with decompensated cirrhosis (6,7).

There were no trials evaluating a programme of physical exercise in patients with liver cirrhosis. Therefore, our previous study was the first in this field. It was a randomized pilot study in which we included 17 compensated cirrhotic patients (8 patients performed a programme of moderate exercise for 12 weeks and 9 were control patients). The results showed a statistically significant increase in muscle mass, effort capacity and quality of life in patients in the exercise programme.

Regarding safety, our study included patients that were compensated. They did not present ascites and they were on variceal bleeding prophylaxis with beta blockers if they had esophageal varices. Therefore, according to previous studies assessing acute exercise in patients with cirrhosis (6,7), we could assume that exercise would not produce undesired effects in our patients. Indeed, no complications of cirrhosis were observed during the study. We observed a significant increase in serum creatinine in the group that performed exercise, although there were not differences in the glomerular filtration rate (GFR) or serum cystatin C. Therefore the increase in serum creatinine was attributed to the increase in muscle mass but not to an impairment in renal function.

Oxidative stress accelerates the progression of liver fibrosis in chronic liver disease of various etiologies (8), it contributes to the development of portal hypertension and hyperdynamic circulation (9,10), and plays an important role in the development of hepatic encephalopathy (11). Exercise can increase oxidative stress (12) and this was one of the risks to be controlled in the previous study. However, we did not observe any increase in the plasma levels of malondialdehyde (MDA) as an index of oxidative stress in patients in the exercise programme.

Finally, cirrhotic patients have a pro-inflammatory state (13) that could be aggravated by exercise (14). Our patients, however, had no increase in serum levels of C-reactive protein (CRP) and proinflammatory cytokines such as interleukin-6 (IL -6) and factor of tumor necrosis alpha (TNF-α) after the exercise programme.

The results of this first pilot study can determine in the future the implementation of exercise programmes as a complementary treatment to improve exercise capacity, muscle mass, quality of life and perhaps prognosis in cirrhotic patients. However, the mentioned results raised several issues of interest that we would like to investigate further.

First, as in the previous study we used rough methods to evaluate exercise capacity and muscle mass (6-minute walk test and anthropometry), we aim to use more accurate methods for assess changes in effort capacity and muscle mass, such as cardiopulmonary exercise test (CPET), CT scan and dual energy X-ray absorptiometry (DXA) (5). Second we want to include a larger number of patients to confirm the efficacy and safety observed in the previous pilot study.

Moreover, it would be interesting to investigate other mechanisms potentially involved in the beneficial effects of exercise in these patients. In this regard, we proposed to analyze changes in glutamine synthetase activity in peripheral blood cells. It is interesting to evaluate whether increased muscle mass leads to an increase in the activity of this enzyme. This would reflect a higher ammonia purification capacity by the muscles, which in turn would be beneficial to reduce the risk of encephalopathy or cognitive impairment in cirrhotic patients (3,15,16).

Another interesting aspect is a more comprehensive analysis of the immune response and oxidative stress and their variations with exercise. We have already commented that cirrhotic patients have a pro-inflammatory state (13) and excessive oxidative stress involved in a worse course of illness (10). It has been suggested that exercise can modulate an excessive inflammatory response and reduce oxidative damage (17,18). These effects could be of particular interest in cirrhotic patients.

Main aims

1. To more accurately evaluate the effect of exercise on effort capacity and muscle mass in patients with cirrhosis.
2. Confirm the efficacy and safety of an exercise program in a larger series of patients with cirrhosis.
3. Explore the mechanisms involved in the beneficial effect of exercise in patients with cirrhosis.
4. Analyze the complications of cirrhosis during the study and follow-up.

Secondary aims

- Analyze changes in the risk of falls.
- Analize changes in inflammatory response and oxidative damage.
- Analyze changes in the expression of glutamine synthetase in peripheral blood cells.
- Analyze variations in the quality of life.

Methodology

Study subjects

Thirty outpatients with compensated liver cirrhosis visited at the Department of Gastroenterology of the Hospital de la Santa Creu i Sant Pau. The study will be conducted at the Hospital de la Santa Creu i Sant Pau.

Inclusion criteria

Patients 18 to 80 years old diagnosed of liver cirrhosis that is currently compensated.

Exclusion Criteria

1. Decompensated cirrhosis.
2. History of bleeding esophageal varices (<3 months).
3. Large esophageal varices without bleeding prophylaxis.
4. Patients with severe comorbidities (heart disease and / or respiratory severe, severe chronic renal failure, etc.).
5. Contraindication for exercise.

6. End-stage cirrhosis as shown by the Model for End-stage Liver Disease (MELD) >25.

7. Life expectancy of less than 6 months.

8. Refusal to participate.

9. Active alcoholism during the year prior to inclusion.

10. Hepatocellular carcinoma or other neoplastic disease at any stage.

Study design

After the baseline multidisciplinary evaluation, patients will be randomized into two groups:

- Group exercise (n=15): will perform an exercise programme that will include relaxation techniques 3 times a week for 12 weeks.
- Control group (n=15): will attend and make relaxation sessions 3 times a week for 12 weeks.

All patients will be evaluated at baseline and at the end of the programme (12 weeks). A follow-up visit will be performed at 24 weeks.

Baseline and 12 week (end of the programme) evaluations

- Medical history and clinical examination, with special emphasis on the detection of complications of cirrhosis and/or exercise, especially encephalopathy, gastrointestinal bleeding, ascites and renal failure during the study and follow-up.
- Anthropometry: weight, height, body mass index, and circumference measurements in the right arm and leg to estimate muscle mass and fat mass. Leg measurements will include two thigh circumferences: upper and lower thigh, measured at one third and at two thirds, respectively, of the line between the trochanter and the upper edge of the kneecap; and mid-thigh skinfold thickness. Measurements in the arm will consist of mid-arm circumference and triceps skinfold thickness, used to calculate mid-arm muscle circumference (19,20).
- Body composition by dual energy X-ray absorptiometry (DXA). DXA will be performed with a Hologic Discovery DXA system® (HOLOGIC, Bedford, MA, USA). The coefficient of variation is 1%. Scan acquisition and the scan analysis will be blindly performed in accordance with ISCD

standards (http://www.iscd.org/documents/2015/06/2015-iscd-adult-official-positions.pdf).

- CT scan of the right thigh to blindly measure muscle volume with a dedicated specific software.
- Functional capacity by cardiopulmonary exercise test (CPET). At the beginning and at the end of the study, participants will perform a stress test on a Schiller STM–55/65 model treadmill using a ramp protocol. The supervisors of CPET will be blinded to the group the patients are assigned to. The initial treadmill speed will be 3 km/h for the first 2 min, increasing 0.3 km/h for each subsequent minute. The initial slope will be zero, increasing 1.4% after the second stage until a maximum of 12% was reached. Twelve-lead ECG (CS-200) recordings will be made and blood pressure taken with a Riester sphygmomanometer. A mask will be used to collect exhaled gases. Using a Ganshorn Power-Cube gas analyser, we will determine the following parameters: oxygen uptake (VO_2) in ml·kg·min, oxygen pulse (PO_2) in ml/beats, carbon dioxide production (VCO_2) in l/min, respiratory quotient (RQ), baseline heart rate (HR) and maximum HR under stress (stress HR), ventilatory anaerobic threshold (VAT) expressed in relation to HR, percentage of VO_2 achieved in the VAT (%VO_2), and exhaled volume in l/min. Baseline and maximum blood pressure will be determined at each stage, and test duration was noted in minutes. The criteria for ending the test will be a lack of increase in VO_2, physical exhaustion, or the reiterated request of the patient to end the test. The criteria for the test maximum will be the appearance of a VO_2 plateau or the appearance of the VAT if the former is lacking.
- Risk of falls will be estimated using the Timed Up&Go test (21,22).
- Blood samples for routine analyses (including liver and renal function) and samples stored for later determination of serum cytokines (TNF-α, IL-6, IL-10, receptor antagonist interleukin-1ra (IL-1ra), expression of TLR2 and TLR4, glutamine synthetase in peripheral blood cells, and MDA, as index of oxidative damage. In total 60 ml of blood will be needed throughout the study.
- Quality of life questionnaire SF-36 (23).

Exercise programme

Patients in the exercise group will perform a 12-week exercise programme, similar to programmes used in other populations, such as patients with chronic heart or respiratory disease (24,25), and to the programme used in patients with cirrhosis in our previous study. The programme will be conducted at the hospital and will consist of 36 one-hour sessions, held on Mondays, Wednesdays and Fridays. Patients will be divided into 2 groups and always led by a physiotherapist. We will measure oxygen saturation, heart rate and blood pressure before, during and at the end of each exercise session. The patient's presence or absence at each session will be recorded.

After a 10-minute warm up, exercise will consist mainly of cycle ergometry and treadmill walking. Cycle ergometry combined with treadmill walking will be performed for 10-15 minutes per session at the beginning of the programme and increasing progressively to 25-30 minutes at the end of the study. Initial treadmill velocity will be calculated and later increased according to patients' tolerance. Patients also will perform resistance exercise for arms using free weights and elastic bands for 5-10 minutes. Throughout the programme, patients will also perform balance, coordination, stretching and relaxation exercises for 10-15 minutes at the end of each session. The intensity of exercise will be increased on the basis of patient tolerance. To prevent variceal bleeding, the programme will not include exercises involving the abdominal muscles or exercises that could increase intra-abdominal pressure. The intensity of the programme are considered moderate, as it was planned that patients work at 60-70% of the maximum heart rate determined using the 220-age formula (26).

Relaxation programme

The relaxation programme will consist of 36 one-hour sessions held Mondays, Wednesdays and Fridays over 12 weeks. Patients in this programme will be included in a single group of 15 patients. Sessions will be led by a physiotherapist trained in relaxation techniques based on sophrology, and they include cephalocaudal muscle relaxation, and breathing, visualization and concentration exercises (27)

Statistical analysis

Baseline characteristics of patients in both groups will be compared using Fisher's exact test for categorical variables and Mann-Whitney test for quantitative variables. The Wilcoxon test will be used to evaluate variations at the end of the study with respect to baseline values in each group. Correlations will be assessed using the Pearson test. A two-sided p value of $p<0.05$ will be considered statistically significant. The sample size has been calculated using previous data on changes in exercise capacity after exercise in patients with cirrhosis. Considering a 30% increase in exercise capacity with an estimated standard deviation of 30%, an alpha error of 0.05, a power of 0.80 and 20% of lost patients, the minimal number of patients needed to detect a significant increase in exercise capacity after the exercise programme is 10.

Study completion

The study will be completed in any of the following circumstances: protocol completion, death, adverse effects (complication of cirrhosis, poor exercise tolerance ...), and at the request of the patient, loss or failure to follow treatment.

Ethical aspects

The protocol will be approved by the Ethics Committee (Comité Ético de Investigación Clínica [CEIC]) at the Hospital de la Santa Creu i Sant Pau. All patients will be informed about their participation in the study and will sign a consent form.

Working plan

Patients will be recruited by Dr. Germán Soriano and Dr. Carlos Guarner from the Department of Gastroenterology. Dr. Ricard Serra-Grima and Maite Doñate from the Department of Cardiology will monitor the exercise programme and the performance of the CPET. Ms. Maria Teresa Torrades, physiotherapist from the Department of Physical Medicine and Rehabilitation, will perform anthropometric measurements and supervise the exercise and relaxation programmes. The physiotherapist Ms. Cristina García-Galcerán will collaborate in the development of the two programmes. Ms. Eva Román, nurse from the

Department of Gastroenterology, will coordinate the development of the study, will obtain and process blood samples, and will perform the quality of life questionnaires. Analytical determinations will be performed at the Laboratory of Biochemistry (Dr. José Rodriguez) and the Laboratory of Immunology (Dr. Cándido Juárez and Dr. Silvia Vidal). Dr. Jaume Llauger from the Department of Radiology will supervise thigh CT scan. DXA will be performed in the Department of Internal Medicine by Ms. Ana Marín and Ms. Sílvia Herrera and will be supervised by Dr. Jorge Malouf. The results will be analyzed by the researchers of the team at the end of the study.

References

1. Bruguera M, Rodés J. Cirrosis hepática compensada. En. Tratamiento de las enfermedades hepáticas y biliares. 2ª ed. Eds. Berenguer J, Bruguera M, García M, Rodrigo L (Asociación Española para el Estudio del Hígado). ELBA, SA. Madrid 2001. págs. 99-104.
2. Caregaro L, Alberino F, Amodio P, et al. Malnutrition in alcoholic and virus-related cirrhosis. Am J Clin Nutr 1996;63:602-609.
3. Córdoba J, Mínguez B. Hepatic encephalopathy. Semin Liver Dis 2008;28:70-80.
4. Ortiz M, Jacas C, Córdoba J. Minimal hepatic encephalopathy: diagnosis, clinical significance and recommendations. J Hepatol 2005;42:S45-S53.
5. Alfonso J, Baeyens JP, Bauer JM, et al. Sarcopenia: European consensus on definition and diagnosis. Age and Aging 2010;39:412-423.
6. Saló J, Ginés A, Anibarro L, et al. Impairment of renal function during moderate physical exercise in cirrhotic patients with ascites: Relationship with the activity of neurohormonal systems. Hepatology 1997;25:1338-1342.
7. García-Pagán JC, Santos C, Barberá JA, et al. Physical exercise increases portal pressure in patients with cirrhosis and portal hypertension. Gastroenterology 1996;111:1300-1306.
8. Parola M, Robino G. Oxidative stress-related molecules and liver fibrosis. J Hepatol 2000;32:141-156.
9. Marley R, Harry D, Arnand R, et al. 8-Isoprostaglandinn F2a, product of lipid peroxidation increases portal pressure in normal and cirrhotic rats. Gastroenterology 1997;112:208-213.
10. Lee KC, Yang YY, Wang YW, et al. Increased plasma malondialdehyde in patients with viral cirrhosis and its relationships to plasma nitric oxide endotoxin, and portal pressure. Dig Dis Sci 2010;55:2077-2085.
11. Jalan R, Kapoor D. Reversal of diuretic-induced hepatic encephalopathy with infusion of albumin but not colloid. Clin Sci 2004;106:467-474.
12. Mergener M, Rosso M, Venzon M, et al. Oxidative stress and DNA damage in older adults that do exercises regularly. Clin Biochemistry 2009;42:1648-1653.
13. Guarner C, Soriano G. Bacterial translocation and its consequences in patients with cirrhosis. Eur J Gastroenterol Hepatol 2005;17:27-31.

14. Pedersen BK, Steensberg A, Fischer C, et al. Exercise and cytokines with particular focus on muscle-derived IL-6. Exerc Immunol Rev 2001;7:18-31.
15. Olde SW, Jalan R, Redhead DN, et al. Interorgan ammonia and amino acid metabolism in metabolically stable patients with cirrhosis and TIPSS. Hepatology 2002;36:1163-1171.
16. Shawcross DL, Wright G, Olde Damink SWM, et al. Role of ammonia and inflammation in minimal hepatic encephalopathy. Metab Brain Dis 2007;22:125-138.
17. Gleeson M. Inmune function in sport and exercise. J Appl Physiol 2007;103:693-699.
18. O'Connor MF, Irwin MR. Links between behavioral factors and inflammation. Clin Pharmacol Ther 2010;87:479-482.
19. Lukaski H. Sarcopenia: assessment of muscle mass. J Nutr 1997;127(5Suppl):994S-997S.
20. Wang J, Thornton JC, Kolesnik S, Pierson RN jr. Anthropometry in body composition. An overview. Ann N Y Acad Sci 2000;904:317-326.
21. Cruz-Jentoft AJ, Baeyens JP, Bauer JM, et al. Sarcopenia: European consensus on definition and diagnosis: Report of the European Working Group on Sarcopenia in Older People. Age Ageing 2010;39:412-423.
22. Viccaro LJ, Perera S, Studenski SA. Is timed up and go better than gait speed in predicting health, function, and falls in older adults? J Am Geriatr Soc 2011;59:887-892.
23. Les I, Doval E, Flavià M, et al. Quality of life in cirrhosis is related to potentially treatable factors. European J Gastroenterol Hepatol 2010;22:221-227.
24. Piña IL, Apstein CS, Balady GJ, et al. Exercise and heart failure: A statement from the American Heart Association Committee on exercise, rehabilitation, and prevention. Circulation 2003;107:1210-1225.
25. Nici L, Donner C, Wouters E, et al. American Thoracic Society/European Respiratory Society statement on pulmonary rehabilitation. Am J Respir Crit Care Med 2006;173:1390-1413.
26. Centers for Disease Control and Prevention. Target heart rate and estimated maximum heart rate. Atlanta, GA, USA, 2011. http://www.cdc.gov/physicalactivity/everyone/measuring/heartrate.html.

27. Caycedo N, Carsí Costas N, Van Rangelrooy K. Sophrology. Rev Enferm 2005;28:30-38.
