# Supplementary material for: Effects of an Exercise Programme on Functional Capacity, Body Composition and Risk of Falls in Patients with Cirrhosis: A Randomized Clinical Trial
Source: PLoS One. 2016 Mar 24;11(3):e0151652. doi: 10.1371/journal.pone.0151652 (PMC4807034; doi:10.1371/journal.pone.0151652)
Supplement: S3 Text — (PDF) [file pone.0151652.s004.pdf]

MECANISMOS IMPLICADOS EN LOS BENEFICIOS DE UN PROGRAMA DE EJERCICIO FÍSICO EN PACIENTES CON CIRROSIS HEPÁTICA

CÓDIGO: 59/2010

Eva Román, Cristina García-Galcerán, Teresa Torrades, Silvia Herrera, Ana Marín, M. Teresa Doñate, Jorge Malouf, Laura Nácher, Ricard Serra-Grima, Carlos Guarner, Juan Cordoba y German Soriano

Servicio de Patología Digestiva, Servicio de Medicina Física y Rehabilitación, Servicio de Cardiología, Servicio de Inmunología, Servicio de Medicina Interna, Hospital de la Santa Creu i Sant Pau. Barcelona.

Resumen

Antecedentes: Los resultados del estudio piloto que hemos realizado en 17 pacientes con cirrosis hepática compensada han mostrado que un programa de ejercicio físico moderado durante tres meses aumenta la masa muscular, la tolerancia al esfuerzo y mejora la calidad de vida sin observarse efectos indeseables. Sin embargo, en este estudio previo evaluamos la masa muscular y la tolerancia al esfuerzo mediante métodos poco precisos, como la antropometría y el test de la marcha de los 6 minutos. No se conocen bien los mecanismos implicados en los beneficios del ejercicio en estos pacientes.

Objetivos principales:

1) Evaluar de una forma más precisa los efectos del ejercicio sobre la capacidad de ejercicio, la masa muscular y la respuesta inflamatoria en pacientes con cirrosis.
2) Confirmar la eficacia y la seguridad de un programa de ejercicio en una serie más amplia de pacientes con cirrosis compensada.

Sujetos: Treinta pacientes con cirrosis hepática compensada.

Intervención: Los pacientes se aleatorizarán en dos grupos: un grupo realizará un programa de ejercicio físico durante 12 semanas a razón de tres días por semana, y el otro será el grupo control que realizará un programa de relajación.

Evaluación: Se realizarán exámenes clínicos y analíticos al inicio y a las 12 semanas (final del estudio). Se analizarán los cambios en la masa muscular mediante técnicas avanzadas (densitometría y scanner), además de la antropometría, así como en la tolerancia al esfuerzo mediante prueba de esfuerzo. Además se evaluarán los cambios en calidad de vida, actividad glutamina sintetasa, daño oxidativo y respuesta inflamatoria. Se analizarán las complicaciones de la cirrosis durante el estudio y en el seguimiento posterior.

Introducción

Una proporción elevada de pacientes cirróticos presenta disminución de la masa muscular, astenia, escasa tolerancia al esfuerzo y disminución de la calidad de vida (1-5), que limitan en mayor o menor grado su actividad normal. Se había sugerido que estos pacientes pueden mejorar si practican ejercicio moderado (1), pero algunos estudios alertaban acerca de los posibles efectos negativos del ejercicio agudo sobre la función renal y la presión portal en los pacientes con cirrosis descompensada (6,7).

Hasta ahora no se habían realizado ensayos que evaluaran un programa de ejercicio físico en pacientes con cirrosis hepática. Por tanto, nuestro estudio previo ha sido el primero en este campo. Se trata de un estudio piloto aleatorizado en el que se incluyeron 17 pacientes cirróticos compensados (8 pacientes realizaron un programa de ejercicio moderado durante 12 semanas y 9 pacientes control). Los resultados mostraron un aumento estadísticamente significativo de la masa muscular, la tolerancia al esfuerzo y la calidad de vida en los pacientes que siguieron el programa de ejercicio.

Respecto a la seguridad, en nuestro estudio los pacientes incluidos estaban compensados, no presentaban ascitis y en el caso de presentar varices esofágicas, estaban en profilaxis de hemorragia por varices con betabloqueantes. Por tanto, según los estudios previos que evaluaban situaciones de ejercicio agudo (6,7), podíamos suponer que el ejercicio no produciría efectos indeseables en nuestros pacientes. Efectivamente, no se observaron complicaciones de la cirrosis durante el estudio ni se registraron cambios en los parámetros hemodinámicos analizados. Sí que se observó un aumento significativo de la creatinina sérica en el grupo que realizó ejercicio, aunque no se observaron diferencias en el filtrado glomerular renal ni en la cistatina C sérica, por lo que este incremento de la creatinina sérica se atribuyó al aumento de masa muscular y no a un deterioro de la función renal.

El estrés oxidativo acelera la progresión de la fibrosis hepática en la enfermedad hepática crónica de diferentes etiologías (8), contribuye al desarrollo de la hipertensión portal y la circulación hiperdinámica (9,10), y juega un papel muy importante en el desarrollo de la encefalopatía hepática (11). El ejercicio físico puede aumentar el estrés oxidativo (12) por lo que éste era uno de los riesgos a controlar en el estudio previo. Sin embargo, no observamos

incremento en los niveles de malondialdehido (MDA) plasmático como índice de estrés oxidativo en los pacientes que siguieron el programa de ejercicio. Finalmente, si bien los pacientes cirróticos presentan un estado proinflamatorio (13) que podría agravarse por el ejercicio (14), nuestros pacientes no presentaron aumento en los niveles séricos de proteína C reactiva (PCR) ni de citoquinas proinflamatorias como interleucina-6 (IL-6) y factor de necrosis tumoral-alfa (TNF-alfa).

Valoramos muy positivamente los resultados de este primer estudio piloto, que puede determinar en un futuro la introducción de los programas de ejercicio como tratamiento complementario para mejorar la capacidad de ejercicio, la masa muscular, la calidad de vida y tal vez el pronóstico en los pacientes cirróticos. Sin embargo, a partir de los resultados comentados se nos plantean diversas cuestiones que creemos de interés seguir investigando.

En primer lugar, en el estudio previo usamos métodos poco precisos para evaluar la capacidad de ejercicio y la masa muscular (el test de la marcha de los 6 minutos y la antropometría). Por tanto, nos planteamos utilizar métodos más precisos para analizar los cambios en la capacidad de ejercicio y la masa muscular, como la prueba de esfuerzo, la densitometría y el scanner (5). En segundo lugar, nos planteamos incluir un mayor número de pacientes que en el estudio previo para confirmar la eficacia y seguridad observadas en dicho estudio.

Por otra parte, sería interesante profundizar en otros mecanismos potencialmente implicados en los efectos beneficiosos del ejercicio en estos pacientes. En este sentido, nos proponemos analizar los cambios en la actividad glutamina sintetasa en células de sangre periférica, ya que sería interesante evaluar si el aumento de la masa muscular produce un incremento en la actividad de esta enzima. Dicho aumento reflejaría una mayor capacidad depurativa del amonio por parte de la musculatura, que a su vez sería beneficioso para disminuir el riesgo de encefalopatía o deterioro cognitivo en los pacientes cirróticos (3,15,16).

Otro aspecto interesante es realizar un análisis más amplio de la respuesta inmune y el estrés oxidativo y sus variaciones con el ejercicio. Ya hemos comentado que los pacientes cirróticos presentan un estado proinflamatorio (13) y un excesivo estrés oxidativo implicados en una peor evolución de su

enfermedad (10). Se ha sugerido que el ejercicio puede modular una respuesta inflamatoria excesiva y disminuir el daño oxidativo (17,18). Estos efectos podrían ser de especial interés en los pacientes cirróticos.

Objetivos principales

1) Evaluar mediante métodos precisos el efecto del ejercicio sobre la tolerancia al esfuerzo y la masa muscular en pacientes con cirrosis.
2) Confirmar la eficacia y seguridad de un programa de ejercicio en una serie más amplia de pacientes con cirrosis.
3) Explorar los mecanismos implicados en los efectos beneficiosos del ejercicio en pacientes con cirrosis.
4) Analizar las complicaciones de la cirrosis durante el estudio y en el seguimiento posterior.

Objetivos secundarios

- Analizar los cambios en el riesgo de caídas.
- Analizar los cambios en la respuesta inflamatoria y el estrés oxidativo.
- Analizar variaciones en la expresión de la glutamina sintetasa en células de sangre periférica.
- Analizar variaciones en la calidad de vida.

Metodología

Sujetos del estudio

Treinta pacientes con cirrosis hepática compensada seleccionados en las consultas externas de los Servicios de Patología Digestiva del Hospital de la Santa Creu i Sant Pau. El estudio se realizará en el Hospital de la Santa Creu i Sant Pau.

Criterios de inclusión

Pacientes de 18 a 80 años, diagnosticados de cirrosis hepática actualmente compensada.

Criterios de exclusión

1. Cirrosis descompensada.

2. Antecedente de hemorragia por varices esofágicas (< 3 meses).
3. Varices esofágicas grandes sin profilaxis.
4. Pacientes con comorbilidades severas (enfermedad cardiaca y/o respiratoria severas, insuficiencia renal crónica severa, etc.).
5. Contraindicación para el ejercicio.
6. Insuficiencia hepática avanzada (Model for End-stage Liver Disease [MELD] <25).
7. Expectativa de vida < 6 meses.
8. Enolismo activo durante el año previo a la inclusión.
9. Hepatocarcinoma u otra enfermedad neoplásica en cualquier estadio.
10. No aceptación a participar en el estudio.

Diseño del estudio

Tras la evaluación basal de carácter multidisciplinar, los pacientes se aleatorizarán en dos grupos:

- Grupo ejercicio (n=15): realizarán un programa de ejercicio 3 veces por semana durante 12 semanas. Al final de cada sesión se les practicarán técnicas de relajación.

- Grupo control (n=15): asistirán 3 veces por semana durante 12 semanas y harán sesiones de relajación utilizando la sofrología como técnica.

A todos los pacientes se les realizará la valoración inicial basal y una valoración al final del programa (12 semanas). Además, habrá una visita de seguimiento a las 24 semanas.

Determinaciones basales y a las 12 semanas (final del programa)

- Historia y exploración clínica con especial énfasis en las complicaciones de la cirrosis y/o del ejercicio durante el estudio y el seguimiento.
- Valoración antropométrica: talla, peso e índice de masa corporal (IMC), medición de perímetros y pliegues subcutáneos en brazo y pierna derechos para estimar la masa muscular y la masa grasa. Las medidas en la pierna incluirán dos circunferencias: muslo superior y muslo inferior, determinadas a un tercio y a dos tercios, respectivamente, de la línea entre el trocánter y el borde superior de la rótula; y el pliegue subcutáneo a nivel de la mitad del muslo medio. Las determinaciones en el brazo consistirán en la

circunferencia y el pliegue subcutáneo en la mitad del brazo, utilizados para calcular la circunferencia muscular del brazo (19,20).

- Composición corporal mediante densitometría mediante Hologic Discovery DXA system® (HOLOGIC, Bedford, MA, USA). El coeficiente de variación es del 1%. La adquisición de scans y los análisis de los mismos se realizará de forma ciega de acuerdo a los ISCD standards (http://www.iscd.org/documents/2015/06/2015-iscd-adult-official-positions.pdf).
- Scanner (tomografía computerizada) para medir de forma ciega el volumen muscular del muslo derecho mediante un software específicamente diseñado para ello.
- Capacidad funcional mediante prueba de esfuerzo. Los participantes realizarán la prueba de esfuerzo en una cinta continua modelo Schiller STM–55/65 mediante protocolo en rampa. Los supervisores de la prueba serán ciegos respecto al grupo asignado. La velocidad inicial de la cinta será de 3 km/h durante los primeros 2 min, aumentando 0.3 km/h en cada minuto posterior. La pendiente inicial será cero, aumentando 1.4% después de la segunda etapa hasta un máximo de 12%. Se realizará una monitorización mediante ECG de 12 derivaciones (CS-200) ya la presión sanguínea se controlará mediante un esfigmomanómetro Riester. Se usará una mascara para recoger los gases exhalados. Mediante un analizador de gases Ganshorn Power-Cube, se determinará: gasto de oxígeno (VO_2) en ml·kg·min, pulso de oxígeno (PO_2) en ml/latido, producción de dióxido de carbono (VCO_2) en l/min, cociente respiratorio (RQ), frecuencia cardíaca basal y máxima, umbral ventilatorio anaerobio (VAT) expresado en relación a la frecuencia cardíaca, porcentage de VO_2 conseguido en la VAT (%VO_2), y volumen exhalado en l/min. Se registrará la presión arterial basal y máxima en cada etapa, así como la duración de la prueba. Los criterios para finalizar la prueba serán la falta de aumento en VO_2, la extenuación física, o la petición reiterada por parte del paciente. Los criterios para el máximo de la prueba serán la aparición de una meseta de VO_2 o la aparición de VAT en ausencia de lo anterior.
- Estimación del riesgo de caídas mediante el Timed Up&Go test (21,22).

- Muestras de sangre para análisis de rutina (incluyendo función hepatica y renal) y muestras almacenadas para posterior determinación de citocinas séricas (TNF-α, IL-6, IL-10, receptor antagonist interleukin-1ra (IL-1ra), expresión de TLR2 y TLR4**,** glutamina sintetasa en células de sangre periférica y MDA, como índice de daño oxidativo. Para ello, serán necesarios unos 60 ml de sangre durante todo el estudio.
- Cuestionario de calidad de vida SF-36 (23).

Programa de ejercicio

Los pacientes de este grupo realizarán un programa de ejercicio de 12 semanas, parecido a los programas utilizados en otras poblaciones, como pacientes con enfermedad crónicas respiratorias o cardíacas (24,25), y al programa utilizado en pacientes con cirrosis en nuestro estudio previo. El programa se realizará en el hospital los lunes, miércoles y viernes durante 12 semanas (36 sesiones). Los pacientes se distribuirán en dos grupos, siempre con la presencia de una fisioterapeuta. Se medirá la saturación de oxígeno, frecuencia cardíaca y presión sanguínea antes, durante y al final de cada sesión de ejercicio. Se registrará la presencia o ausencia de cada paciente en cada sesión.

Después de 10 min de calentamiento, el ejercicio consistirá sobre todo en bicicleta estática y tapiz rodante. La bicicleta estática se combinará con el tapiz rodante durante 10-15 min por sesión al inicio del programa, aumentando progresivamente hasta 25-30 min al final. La velocidad inicial del tapiz rodante se calculará y se aumentará de acuerdo a la tolerancia del paciente. Los pacientes llevarán a cabo ejercicios de resistencia de los brazos con pesas y bandas elásticas durante 5-10 min. Los pacientes también realizarán ejercicios de equilibrio, coordinación, estiramientos y relajación durante 10-15 min al final de cada sesión. La intensidad del ejercicio aumentará en base a la tolerancia de cada paciente. Se evitarán ejercicios que comprometan la musculatura abdominal o que aumenten la presión intra-abdominal, con la finalidad de prevenir la aparición de una de las complicaciones por aumento de la presión portal (hemorragia digestiva por la rotura de varices esofágicas). La intensidad del programa se considera moderada, ya que los pacientes trabajarán al 60-

70% de la frecuencia cardíaca máxima determinada por la fórmula 220-edad (26).

Programa de relajación

El otro grupo del estudio seguirá un programa de relajación de 36 horas, una hora cada lunes, miércoles y viernes durante 12 semanas. Los 15 pacientes de este programa se incluirán en un solo grupo. Las sesiones serán dirigidas por una fisioterapeuta entrenada en técnicas de relajación. El programa se basa en la sofrología e incluye ejercicios de relajación muscular cráneo-caudal, respiración, visualización y concentración (27).

Análisis estadístico

Se compararán las características basales de los dos grupos mediante el test exacto de Fisher para variables cualitativas y el test de Mann-Whitney para variables cuantitativas. El test de Wilcoxon se utilizará para analizar los cambios en las variables cuantitativas al final del estudio respecto al inicio en los dos grupos. Las correlaciones se evaluarán mediante el test de Pearson. Un valor bidireccional de $p<0.05$ se considerará estadísticamente significativo. El cálculo de la muestra se ha realizado en base a datos previos sobre cambios en la capacidad de ejercicio en pacientes con cirrosis después de realizar un programa de ejercicio. Considerando un aumento del 30% en la capacidad de ejercicio con una desviación estándar estimada del 30%, un error alfa del 0.05, un error beta del 0.80 y un 20% de pérdidas, el número mínimo de pacientes necesario para detectar un aumento significativo en la capacidad de ejercicio después de un programa de ejercicio sería de 10.

Finalización del estudio

El estudio finalizará en cualquiera de las siguientes circunstancias: finalización del tratamiento, fallecimiento, efectos adversos (complicación de la cirrosis, mala tolerancia al ejercicio...), a petición del paciente, pérdida de seguimiento o incumplimiento del tratamiento.

Aspectos éticos

El protocolo será sometido para su aprobación por el Comité Ético de Investigación Clínica (CEIC) del Hospital de la Santa Creu i Sant Pau. Todos los pacientes serán debidamente informados sobre su participación en el estudio y firmarán una hoja de consentimiento.

Plan de Trabajo

Los pacientes serán reclutados por los Dres. Germán Soriano y Carlos Guarner. Los Dres. Ricard Serra-Grima y Maite Doñate del Servicio de Cardiología, realizarán la evaluación física inicial y final y supervisarán el programa de ejercicio y las pruebas de esfuerzo. La Sra. Mª Teresa Torrades, fisioterapeuta del Servicio de Medicina Física y Rehabilitación, realizará las medidas antropométricas y supervisará las sesiones de ejercicio y relajación. La fisioterapeuta Sra. Cristina García-Picart colaborará en dichas sesiones. La Sra. Eva Román, enfermera del Servicio de Patología Digestiva, coordinará el estudio, obtendrá y procesará las muestras de sangre y realizará los cuestionarios de calidad de vida. Las determinaciones analíticas se realizarán en el Laboratorio de Bioquímica del Hospital de la Santa Creu i Sant Pau (Dr. José Rodríguez) y el Laboratorio de Inmunologia (Dres. Cándido Juárez y Silvia Vidal). El Dr. Jaume Llauger del Servicio de Radiología supervisará los scanner de los pacientes. Las densitometrías se realizarán en el Servicio de Medicina Interna por las Sras. Ana Marín y Sílvia Herrera y serán supervisadas por el Dr. Jorge Malouf. Los resultados serán analizados por los investigadores del equipo al finalizar el estudio.

Bibliografía

1. Bruguera M, Rodés J. Cirrosis hepática compensada. En. Tratamiento de las enfermedades hepáticas y biliares. 2ª ed. Eds. Berenguer J, Bruguera M, García M, Rodrigo L (Asociación Española para el Estudio del Hígado). ELBA, SA. Madrid 2001. págs. 99-104.
2. Caregaro L, Alberino F, Amodio P, et al. Malnutrition in alcoholic and virus-related cirrhosis. Am J Clin Nutr 1996;63:602-609.
3. Córdoba J, Mínguez B. Hepatic encephalopathy. Semin Liver Dis 2008;28:70-80.
4. Ortiz M, Jacas C, Córdoba J. Minimal hepatic encephalopathy: diagnosis, clinical significance and recommendations. J Hepatol 2005;42:S45-S53.
5. Alfonso J, Baeyens JP, Bauer JM, et al. Sarcopenia: European consensus on definition and diagnosis. Age and Aging 2010;39:412-423.
6. Saló J, Ginés A, Anibarro L, et al. Impairment of renal function during moderate physical exercise in cirrhotic patients with ascites: Relationship with the activity of neurohormonal systems. Hepatology 1997;25:1338-1342.
7. García-Pagán JC, Santos C, Barberá JA, et al. Physical exercise increases portal pressure in patients with cirrhosis and portal hypertension. Gastroenterology 1996;111:1300-1306.
8. Parola M, Robino G. Oxidative stress-related molecules and liver fibrosis. J Hepatol 2000;32:141-156.
9. Marley R, Harry D, Arnand R, et al. 8-Isoprostaglandinn F2a, product of lipid peroxidation increases portal pressure in normal and cirrhotic rats. Gastroenterology 1997;112:208-213.
10. Lee KC, Yang YY, Wang YW, et al. Increased plasma malondialdehyde in patients with viral cirrhosis and its relationships to plasma nitric oxide endotoxin, and portal pressure. Dig Dis Sci 2010;55:2077-2085.
11. Jalan R, Kapoor D. Reversal of diuretic-induced hepatic encephalopathy with infusion of albumin but not colloid. Clin Sci 2004;106:467-474.
12. Mergener M, Rosso M, Venzon M, et al. Oxidative stress and DNA damage in older adults that do exercises regularly. Clin Biochemistry 2009;42:1648-1653.
13. Guarner C, Soriano G. Bacterial translocation and its consequences in patients with cirrhosis. Eur J Gastroenterol Hepatol 2005;17:27-31.

14. Pedersen BK, Steensberg A, Fischer C, et al. Exercise and cytokines with particular focus on muscle-derived IL-6. Exerc Immunol Rev 2001;7:18-31.
15. Olde SW, Jalan R, Redhead DN, et al. Interorgan ammonia and amino acid metabolism in metabolically stable patients with cirrhosis and TIPSS. Hepatology 2002;36:1163-1171.
16. Shawcross DL, Wright G, Olde Damink SWM, et al. Role of ammonia and inflammation in minimal hepatic encephalopathy. Metab Brain Dis 2007;22:125-138.
17. Gleeson M. Inmune function in sport and exercise. J Appl Physiol 2007;103:693-699.
18. O'Connor MF, Irwin MR. Links between behavioral factors and inflammation. Clin Pharmacol Ther 2010;87:479-482.
19. Lukaski H. Sarcopenia: assessment of muscle mass. J Nutr 1997;127(5Suppl):994S-997S.
20. Wang J, Thornton JC, Kolesnik S, Pierson RN jr. Anthropometry in body composition. An overview. Ann N Y Acad Sci 2000;904:317-326.
21. Cruz-Jentoft AJ, Baeyens JP, Bauer JM, et al. Sarcopenia: European consensus on definition and diagnosis: Report of the European Working Group on Sarcopenia in Older People. Age Ageing 2010;39:412-423.
22. Viccaro LJ, Perera S, Studenski SA. Is timed up and go better than gait speed in predicting health, function, and falls in older adults? J Am Geriatr Soc 2011;59:887-892.
23. Les I, Doval E, Flavià M, et al. Quality of life in cirrhosis is related to potentially treatable factors. European J Gastroenterol Hepatol 2010;22:221-227.
24. Piña IL, Apstein CS, Balady GJ, et al. Exercise and heart failure: A statement from the American Heart Association Committee on exercise, rehabilitation, and prevention. Circulation 2003;107:1210-1225.
25. Nici L, Donner C, Wouters E, et al. American Thoracic Society/European Respiratory Society statement on pulmonary rehabilitation. Am J Respir Crit Care Med 2006;173:1390-1413.
26. Centers for Disease Control and Prevention. Target heart rate and estimated maximum heart rate. Atlanta, GA, USA, 2011. http://www.cdc.gov/physicalactivity/everyone/measuring/heartrate.html.

27. Caycedo N, Carsí Costas N, Van Rangelrooy K. Sophrology. Rev Enferm. 2005;28:30-38.
