# Supplementary material for: Effects of an Exercise Programme on Functional Capacity, Body Composition and Risk of Falls in Patients with Cirrhosis: A Randomized Clinical Trial
Source: PLoS One. 2016 Mar 24;11(3):e0151652. doi: 10.1371/journal.pone.0151652 (PMC4807034; doi:10.1371/journal.pone.0151652)
Supplement: S4 Text — (PDF) [file pone.0151652.s005.pdf]

HOSPITAL DE LA SANTA CREU I SANT PAU
UNIVERSITAT AUTÒNOMA DE BARCELONA

Sant Antoni Ma Claret, 167 · 08025 Barcelona
Tel. 93 291 90 00 · Fax 93 291 94 27
e-mail: santpau@santpau.cat
www.santpau.cat

COMITÉ ÉTICO DE INVESTIGACIÓN CLÍNICA

TÍTULO: Mecanismos implicados en los beneficios de un programa de ejercicio físico en pacientes con cirrosis hepática		
CÓDIGO: **59/2010**	IP: **Dr. G. Soriano**	SERVICIO: **Patología Digestiva**

Doña **Milagros Alonso Martínez**, Secretaria del Comité Ético de Investigación Clínica del Hospital de la Santa Cruz y San Pablo,

CERTIFICA:

Que en su reunión de fecha 28 de Septiembre de 2010 este Comité ha analizado el proyecto de investigación de referencia y considera que se ajusta a las disposiciones vigentes.

Por ello, ha acordado informar favorablemente sobre su realización.

Y para que así conste, firma el presente en Barcelona, a 30 de Septiembre de 2010.

FUNDACIÓ DE GESTIÓ SANITÀRIA DE L'HOSPITAL DE LA SANTA CREU I SANT PAU
COMITÈ ÈTIC D'INVESTIGACIÓ CLÍNICA

Dra. Milagros Alonso Martínez

Fundació de Gestió Sanitària de l'Hospital de la Santa Creu i Sant Pau · NIF G-59.780.494
CSB Consorci Sanitari de Barcelona
4000751
